# MicroRNA expression in bone marrow-derived human multipotent Stromal cells

**DOI:** 10.1186/s12864-017-3997-7

**Published:** 2017-08-11

**Authors:** Ian H. Bellayr, Abhinav Kumar, Raj K. Puri

**Affiliations:** Tumor Vaccines and Biotechnology Branch, Division of Cellular and Gene Therapies, Center for Biologics and Evaluation Research, US Food and Drug Administration, Silver Spring, MD USA

**Keywords:** Multipotent Stromal cells, MicroRNA expression, Microarray

## Abstract

**Background:**

Multipotent stromal cells (MSCs) are being studied in the field of regenerative medicine for their multi-lineage differentiation and immunoregulatory capacity. MicroRNAs (miRNAs) are short non-coding RNAs that are responsible for regulating gene expression by targeting transcripts, which can impact MSC functions such as cellular proliferation, differentiation, migration and cell death. miRNAs are expressed in MSCs; however, the impact of miRNAs on cellular functions and donor variability is not well understood. Eight MSC lines were expanded to passages 3, 5 and 7, and their miRNA expression was evaluated using microarray technology.

**Results:**

Statistical analyses of our data revealed that 71 miRNAs out of 939 examined were expressed by this set of MSC lines at all passages and the expression of 11 miRNAs were significantly different between passages 3 and 7, while the expression of 7 miRNAs was significantly different between passages 3 and 5. The expression of these identified miRNAs was evaluated using RT-qPCR for both the first set of MSC lines (*n* = 6) and a second set of MSC lines (*n* = 7) expanded from passages 4 to 8. By RT-qPCR only 2 miRNAs, miR-638 and miR-572 were upregulated at passage 7 compared to passage 3 in the first set of MSC lines by 1.71 and 1.54 fold, respectively; and upregulated at passage 8 compared to passage 4 in the second set of MSC lines, 1.35 and 1.59 fold, respectively.

**Conclusions:**

The expression of miR-638 and miR-572 can distinguish MSCs from two different passages of cell culture. These results may be useful in establishing critical quality attributes of MSCs and determining whether changes in these two miRNAs impact cellular functions.

**Electronic supplementary material:**

The online version of this article (doi:10.1186/s12864-017-3997-7) contains supplementary material, which is available to authorized users.

## Background

MicroRNAs (miRNA) are small non-coding RNAs with a typical sequence length ranging from 17 to 22 nucleotides. Since their initial discovery in *Caenorhabditis elegans*, thousands more miRNAs have been discovered in a number of different species, including animals, plants, and viruses [1–3]. miRNAs are typically located within the intergenic and intronic regions of genes where they are transcribed by RNA polymerase II in the nucleus to generate hairpin pri-miRNAs [4, 5]. The pri-miRNAs undergo further processing by the Drosha enzyme, shortening them to form pre-miRNAs, which are subsequently transported to the cytoplasm by exportin 5 [6, 7]. With the use of the Dicer enzyme, the hairpin loops are cleaved leaving two separate strands, one of which is the mature miRNA. The mature form of the miRNA can bind to a target gene, temporarily repressing its expression or terminating expression by degradation of the transcript [7–9]. Through these two mechanisms, miRNAs are believed to be involved in a number of processes such as cell proliferation, differentiation, apoptosis and cell migration [10–15].

Ongoing research is revealing an ever increasing repertoire of miRNAs, from tissues, blood, urine or saliva, which may be used as biomarkers for cancer or infectious diseases [12, 16–21]. In addition, the role of miRNA expression in the field of stem cell research is being realized [22–24]. Multipotent stromal cells (MSCs), also known as mesenchymal stem cells, are highly studied for a variety of applications and derived from a variety of tissues [25–27]. As MSCs are highly valued for their regenerative potential to treat traumatic injuries or diseases, researchers are investigating how miRNAs manipulate and drive their behavior [28, 29]. Applying a systems biology approach, a group of 7 scientists in our division (“MSC Consortium”) are characterizing human bone-marrow derived MSCs for their phenotype, cell size, gene expression, protein expression, epigenetics, and morphology in order to identify critical quality attributes of MSCs and determine cellular attributes that can be linked to functional properties such as differentiation, immunosuppression etc. [30–36]. This group has shown that MSCs are a heterogeneous cell population and display functional differences based on donor and cell passaging [30–41]. Currently, no comprehensive information is available on the miRNA expression in human MSCs and whether the miRNA expression changes as a result of cell culture.

With most MSC-based products, cellular expansion is a crucial manufacturing step to generate a sufficient quantity of cells for therapeutic use. Our previous work identified gene markers that may be used to assess cellular aging through MSC expansion [30]. These genes may serve as potential markers of MSC quality as certain attributes of MSCs such as cell size, proliferation, karyotypic abnormalities, and differentiation assays including colony forming units, adipogenesis and osteogenic mineralization have been shown to exhibit changes with expansion [31, 33, 34, 37, 38, 41]. The objectives of the current study are to identify commonly expressed miRNAs in bone marrow-derived MSCs from different donors, evaluate the heterogenic miRNA expression in MSCs, and identify miRNAs that exhibit changes with cellular passage.

## Methods

### Cultivation and expansion of human bone marrow-derived MSCs

A total of 8 human bone marrow-derived MSCs from 8 donors were purchased from ALLCELLS and Lonza at passage 1 (Additional file 1: Table S1). By flow cytometry, all MSCs revealed positive expression for CD29, CD44, CD73, CD90, CD105, and CD166 [31, 38]. MSCs were expanded in medium containing alpha-MEM (Invitrogen), 10% FBS (Atlanta Biologics), 1% L-glutamine (Invitrogen), and 1% penicillin G and streptomycin sulfate (Invitrogen) under a humidified atmosphere of 5% CO_2_ at 37 °C as previously reported [31]. Upon reaching 80% confluency, MSCs were detached from the flask using 0.25% trypsin/1 mM EDTA solution (Invitrogen) and replated at 60 cells/cm^2^. MSCs were expanded to passage 7. Only two donor’s MSCs, 127756 and PCBM1655 did not reach passage 7. The second set of MSCs (*n* = 7) and mesoderm cells lines (*n* = 4) were expanded from passage 4 to 8 in marrow stromal cell growth medium from Cell Applications (San Diego, CA) as described in Bellayr et al. (2016) [37]. Cancer cell lines were purchased from ATCC (Manassas, VA) and cultured according to the manufacturer’s instructions.

### Total RNA preparation

Total RNA isolation was completed on cell samples at either early (P3/P4) or later passage (P7/P8) using the Absolutely RNA miRNA kit from Agilent Technologies (Santa Clara, CA). NTERA-2 (NT2), OVCAR5, SKOV3 and HS766T cells grow indefinitely; therefore no passage number was assigned. Cell lines OVCAR5, SKOV3, and HS766T, were only used in RT-qPCR experiments. The Agilent 2100 bioanalyzer was used to evaluate the RNA integrity number (RIN) for all cellular samples. The total RNA concentration was measured using a Nanodrop 1000 spectrophotometer (Wilmington, DE).

### Microarray hybridization

Sample preparation and hybridization of Agilent Technologies human miRNA microarrays was performed according to the manufacturer’s instructions using Agilent technologies G4471 kit. Briefly, labeling spike-in and Hyp spike-in solutions were diluted, aliquoted and stored at -80 °C until further use. Total RNA of 100 ng was added to the calf intestinal alkaline phosphatase master mix and heated at 37 °C for 30 min for dephosphorylation. Afterward, 100% DMSO was added to samples and heated at 100 °C for 5 min to denature them. Finally, the ligation master mix with Cy3 was combined with the cocktail and incubated at 16 °C for 2 h.

After incubation, the samples were purified using Micro Bio-Spin P-6 gel columns [Bio-Rad]. The remainder of the sample was dried using a vacuum concentrator at 50 °C for approximately 1 h or until the samples were completely dried. The dried samples were then resuspended in nuclease-free water, mixed gently with the 10X GE blocking agent, 2X Hi-RPM hybridization buffer and Hyb spike-in, and incubated at 100 °C for 5 min. Each sample was loaded individually into a separate chamber of an 8 chamber clean gasket slide. Then the miRNA microarray was placed on top and secured with a metal clamp. Assembled slide chambers were placed in the hybridization oven set to 55 °C with a rotator speed of 20 rpm for 20 h. Following miRNA hybridization, slides were washed in the gene expression wash buffers, 1 and 2, purchased from Agilent. All samples from the first set of 8 MSC donors at each passage were run in triplicate using a randomized block design. Total RNA from one cancer cell line, NT2 and universal human reference RNA (from 10 cell lines, Agilent Technologies, #740000) (Additional file 2: Table S2) were also run in duplicate for comparison with MSCs at P3.

### Data acquisition and analysis

The DNA microarray scanner with surescan high-resolution technology (Agilent Technologies) was used to capture images of the microarray slides. Slides were scanned at 446 nm for the Cy3 dye and a resolution of 2 μm double pass. The spots were annotated through the Agilent Feature Extraction (AFE) 11.0 software. All methods and analyses used in this study were implemented in JMP Genomics software by SAS, which included normalization, principle component analysis, clustering, box plots, statistics and application of a 5% false discovery rate. Two processed signals were considered for analysis: 1) AFE - total gene signal (TGS) normalized to the 75th percentile; and 2) quantile normalization of the raw signal without background correction (See Additional file 3: and Additional file 4: Figure S1). For the AFE-TGS method, arrays were normalized according to López-Romero et al. [42]. Based on the literature regarding miRNA microarray normalization and the results presented, quantile normalization was used for further subsequent downstream analyses [43, 44]. miRNA microarray data files have been uploaded into the public repository, Gene Expression Omnibus (GSE87291). It should be noted that the microarrays 8F3560_P7_1 and PCBM1641_P5_1 were removed from the set for further analysis as they were determined to be outliers because the background signal was extremely elevated.

After quantile normalization of all data files, filtering and statistical analysis were performed on the 2686 sequences representing 939 miRNAs. The 2686 sequences were filtered by the within chip technical variability, between chip technical variability, biological variability and magnitude of expression level using recommended methods [30, 45–47]. For the within chip technical variability, the standard deviation was calculated per sequence per array, where the number of sequence replicates varied between 4 and 8 replicates. The median standard deviation of the 61 arrays was termed the within chip technical variability per sequence. Sequences whose differences between passage 3 and 7 was less than the within chip technical variable cutoff were eliminated. The between chip technical variability, biological variability and magnitude of expression was calculated according to Bellayr et al. (2014) using passages 3, 5 and 7 to remove additional miRNA sequences [30]. Following the filtering steps, a repeated measures ANOVA model with compound symmetry correlation structure was performed on the 21 miRNA sequences for the MSC donors that grew to passage 7. For multiplicity adjustment, the Benjamini-Hochberg method was applied with an adjusted *p*-value less than 0.05 for all pairwise comparisons between passages [48].

Comparable methods were used for comparing miRNA expression between MSCs and the cancer samples. An unequal sample size, equal variance t-test was performed to compare the MSC samples from 8 donors at passage 3 to the 2 cancer samples.

To determine the number of miRNAs commonly expressed in all MSC donors above the limit of detection, a background cutoff was calculated from the mean of the 434 negative controls (probes spotted on the microarrays for optimal background-subtraction with the AFE software) plus 1 standard deviation for a specific donor/passage. All miRNA sequences whose median expression level of the technical replicates that were greater than this cutoff were considered expressed for the donor/passage. If the miRNA sequence was observed to be expressed across all donors/passages (61 arrays), then it was considered to be consistently expressed in all 8 MSC donor samples.

### RT-qPCR

RT-qPCR was performed on all MSC and non-MSC lines for 30 different miRNAs (19 to evaluate their magnitude of expression and 11 miRNAs to evaluate differences in cellular passaging) with positive/negative controls (miRTC, PPC) were purchased from Qiagen (Valencia, CA). This technique was used to evaluate miRNA expression between passage 3 and 7 in the first set of MSCs and passage 4 and 8 in the second set of MSCs. Reverse transcription was performed using a total of 1 ng per sample according to the manufacturer’s instructions using the 5× iScript cDNA synthesis kit. Quantitative PCR was performed per the manufacturer’s instructions using SsoAdvanced universal SYBR green supermix. The reaction protocol was 15 s at 95 °C, 30 s at 55 °C and 30 s at 70 °C for 40 cycles. Multiple samples were evaluated in duplicate and triplicate to calculate the variability of the selected primers. miRNAs were normalized using the geometric mean of 5 targets (SNORD61, SNORD68, SNORD72, SNORD95 and SNORD96A).

## Results

### miRNA expression by MSCs

**Fig. 1:**
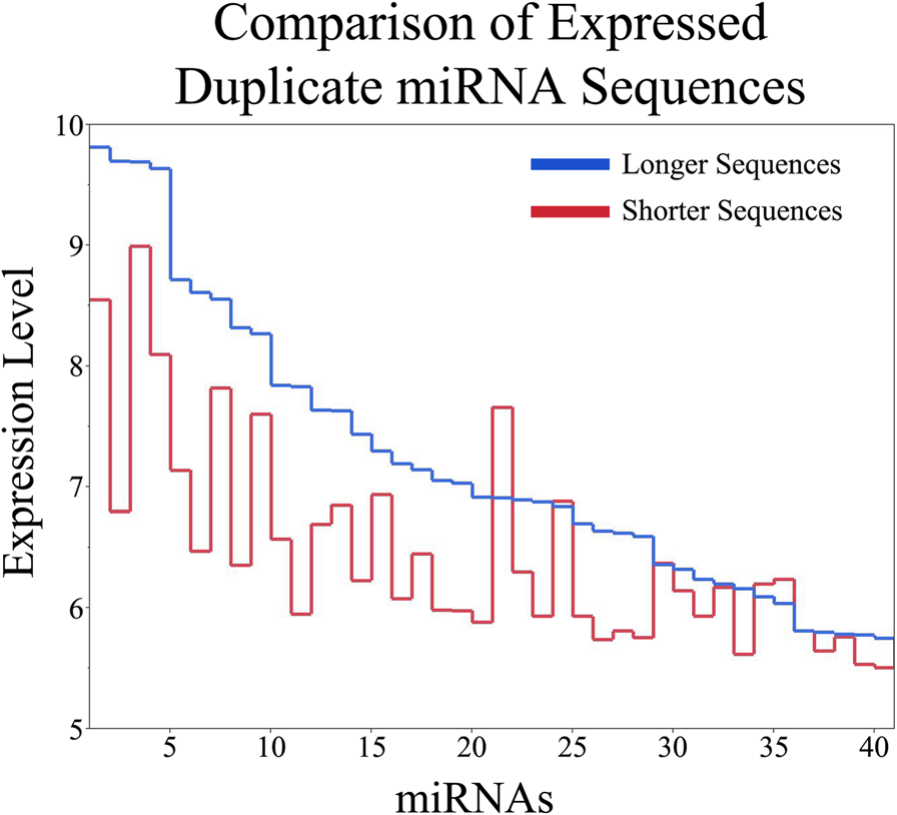
Comparison of the magnitude of expression of short and long sequences of expressed duplicate miRNAs

Since miRNA expression was evaluated using a single color fluorescent probe, we were capable of determining the number of miRNAs that were commonly expressed among all 8 donors’ MSCs across all passages. This was performed by calculating a background cutoff expression value for the 21 different samples from the negative controls (mean + 1 standard deviation) for a given donor/passage. For a specific sequence, if the median expression level across technical replicates of a given donor/passage was greater than the background cutoff and this was true for all 21 donor/passage samples, then the sequence was determined to be expressed in all MSC donors. However, if the median expression across technical replicates was less than at least one background cutoff, then the miRNA sequence was not considered to be commonly expressed by all MSC donors. After applying this method for the entire miRNA dataset, a total of 123 miRNA sequences were found to be commonly expressed in all donors and passages, where 11 of these miRNA sequences were determined to be either a tRNA or rRNA according to miRBase (Additional file 5: Table S3). Furthermore, 71 miRNAs were observed to be expressed in all MSC donors with median expression values ranging from 5.506 to 9.817. Forty-one of the 71 miRNAs had duplicate sequences that varied by a single base pair. In evaluating the expression level of the shorter sequences to the longer sequences for duplicates, statistical significance was found (using a paired t-test, *p* < 0.01) such that the longer sequences had a 1.67 fold increase in expression over shorter sequences (Fig. 1).

**Fig. 2:**
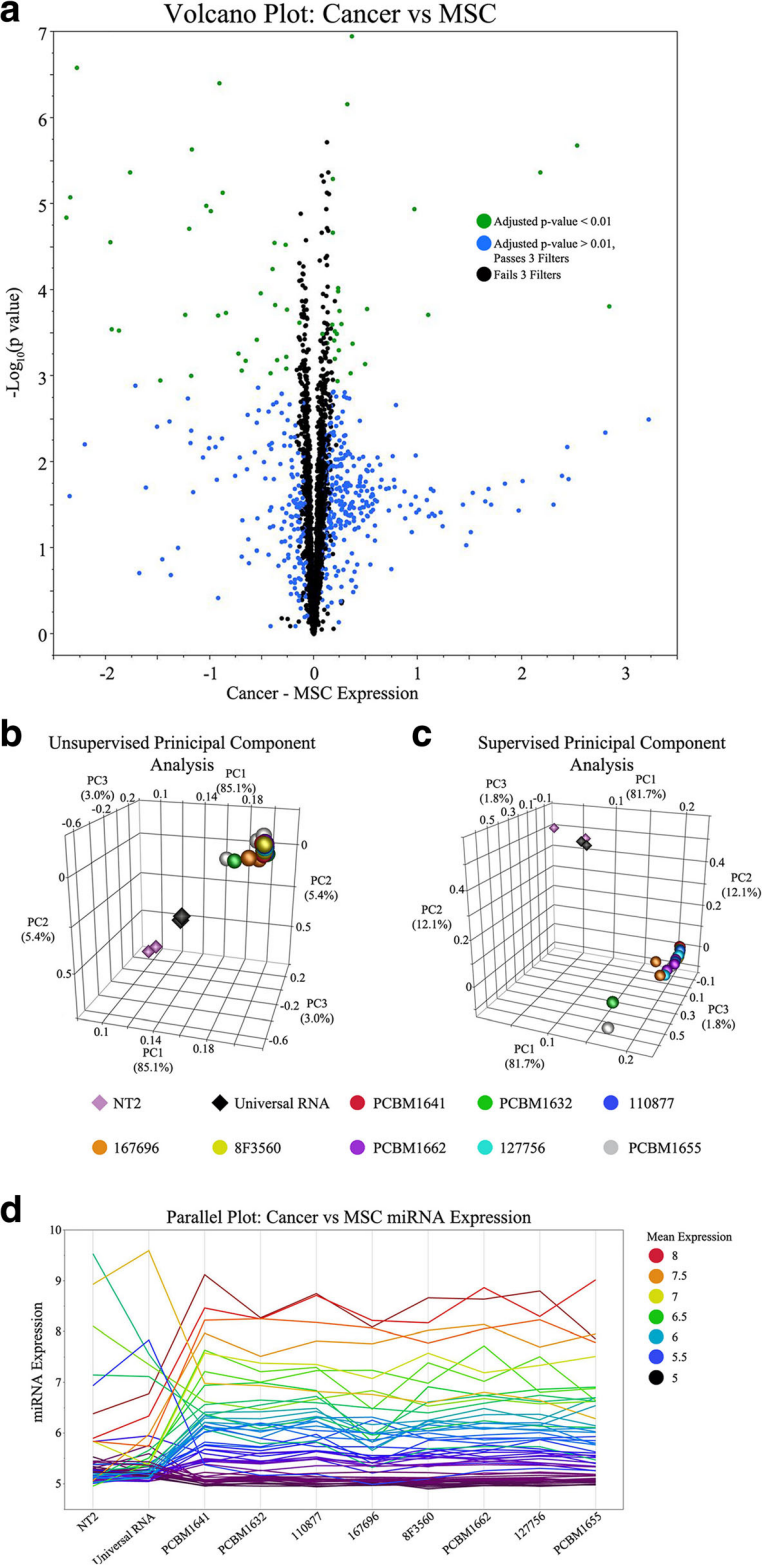
**a** Volcano plot of the difference between cancer and MSC miRNA expression versus the –Log_10_(*p*-value). Green circles (60) indicate the number of statistically significant miRNA sequences. Principal component analyses of the **b** unsupervised 2686 different miRNA sequences prior to analysis; and **c** supervised 60 statistically significant miRNAs. **d** Parallel plot of the significant miRNAs expression levels across all samples. Mean expression is calculated from all 10 samples

To evaluate the potential of these analytical methods to detect differences in miRNA expression between MSC and non-MSCs, a statistical analysis was performed comparing two distinctly different populations of samples; MSCs from 8 donors at passage 3 to 2 cancer samples, NT2 and universal human reference RNA. Using the quantile normalized datasets of 28 miRNA microarrays, a within chip variability and between chip variability cutoff was calculated for each of the 2686 miRNA sequences as well as the mean absolute difference between the cancer and MSC samples. In comparisons where either of the variability cutoffs was greater than the mean absolute difference between the cancer and MSC samples, miRNAs were removed from the dataset (Additional file 6: Figure S2A–B). A total of 1187 miRNA sequences that passed both these filters, were subjected to a magnitude of expression filter where the background cutoff (of the negative controls) was calculated as 5.11 ± 0.09 (Additional file 6: Figure S2C). Following the application of this filter, 491 miRNA sequences remained. Using an unequal sample sizes, equal variances t-test with an adjusted *p*-value of 0.01, 60 miRNA sequences were found to be statistically significant (*p* < 0.01) between MSCs at passage 3 and the cancer samples (Fig. 2a). Out of the significant miRNA sequences, 14 miRNAs were duplicate sequences differing by one base pair and one sequence is considered an rRNA according to miRBase, leaving a total of 45 miRNAs that are significant between the two groups (Additional file 7: Table S4). Small significant differences between MSCs and the cancer samples could be detected as comparable to the significant differences detected between passages of MSCs, however, fold changes as large as 7.16 were observed. In visualizing the multi-dimensional data set, a distinct separation was observed by unsupervised principal component analysis between the clustered MSC samples and the two cancer samples (Fig. 2b). There was some separation between the two cancer samples as the technical replicates clustered closely together. After supervised principal component analysis using the significant miRNA sequences, both cancer samples are more closely clustered together and the degree of separation between them and the MSCs is much greater (Fig. 2c). The magnitude in the differences between NT2 and the universal RNA versus the MSCs at passage 3 is further illustrated by a parallel plot (Fig. 2d). This plot not only highlights the differences in expression between two cancer cell samples and MSCs, but also visualizes the heterogenic miRNA expression among MSCs derived from different donors. The miRNA expression of these 45 miRNAs show varying expression of magnitude deviating from the calculated mean expression across these 10 samples.

### Differentially expressed miRNAs

**Fig. 3:**
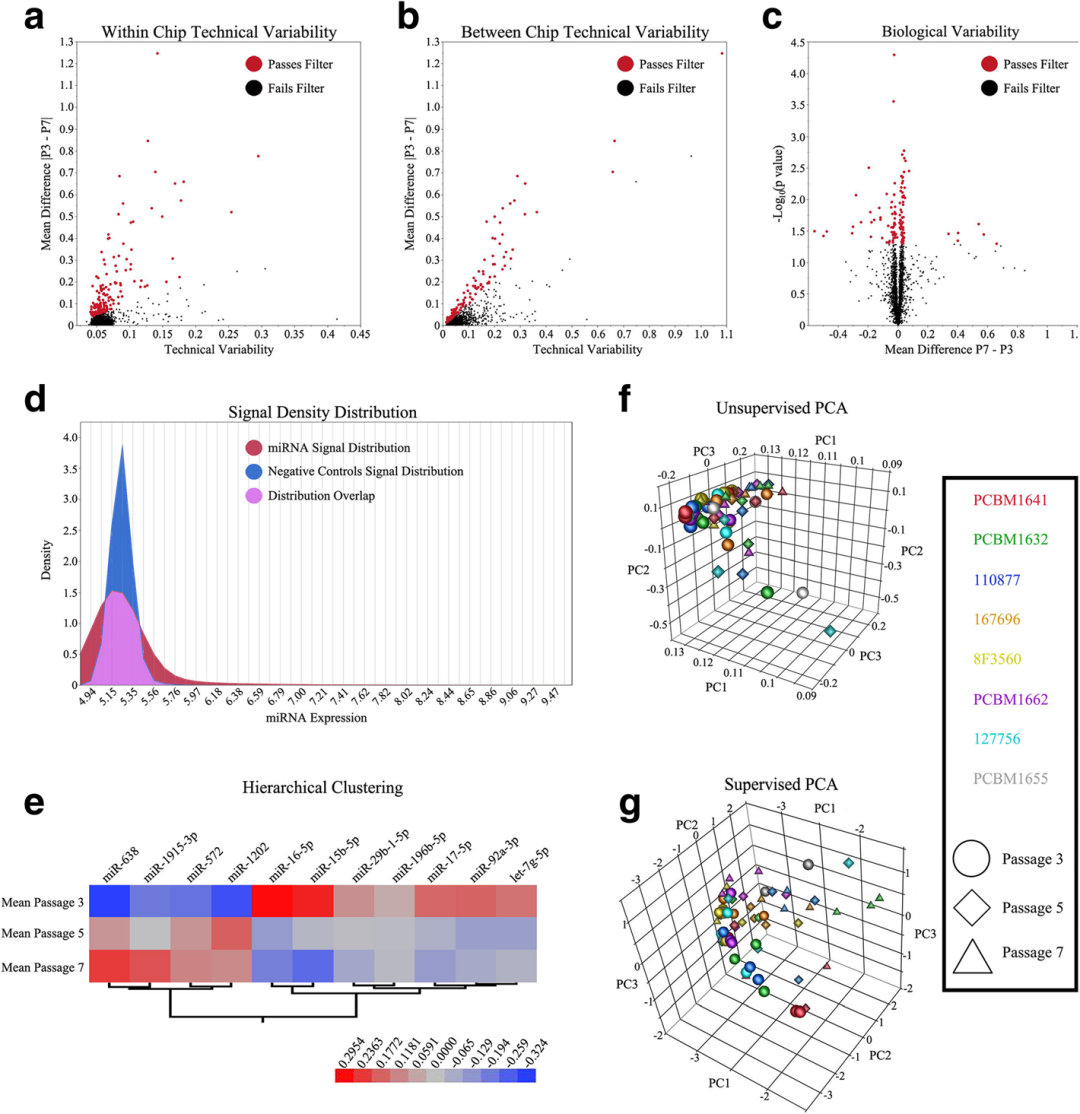
The mean absolute difference between passage 3 and 7 versus the different technical variability cutoffs of **a** within chip; and **b** between chips. **c** Volcano plot of the mean difference between passage 7 and 3 versus –Log_10_(*p*-value) representing the biological variability. Red circles indicate the number of miRNA sequences that passed the biological variability filter (*p*-value <0.05). **d** Signal density distribution of the miRNA signals and the negative controls. **e** Hierarchical clustering heatmap of the significant miRNAs expression levels at passages 3, 5, and 7. Principal component analyses of the **f** unsupervised 2686 different miRNA sequences prior to analysis; and **g** supervised 12 statistically significant miRNA sequences

Another objective of the study was to identify miRNA markers that could be indicative of passaged MSCs in culture after extended expansion. Since all samples were hybridized in triplicate, within chip and between chip technical variability cutoffs were calculated and used to eliminate miRNA sequences where differences between passages were not measurable. miRNA sequences where the absolute difference in expression between passage 3 and 7 was less than the technical variability cutoffs were removed from the dataset as they could not accurately be measured. Applying the within chip and between chip filters to the dataset, 86 of the 2686 miRNA sequences passed (Fig. 3a–b).

A biological variability cutoff and magnitude of expression cutoff was further used to eliminate undetectable differences in miRNAs. Those miRNAs with highly variable expressions between passage 3 and 5 or passage 5 and 7 would not be suitable candidates, thus using paired t-tests between the MSCs donors at passage 3 and 7 with an alpha level above 0.05, 62 miRNA sequences were removed from the dataset (Fig. 3c). For the magnitude of expression filter, a cutoff was determined from the distribution of 434 negative controls across all arrays with a mean of 5.29 ± 0.11 (Fig. 3d). Sequences where the mean expression at passages 3, 5 and 7 were less than the negative controls cutoff were removed, leaving a total of 21 miRNA sequences. After passing the stringent acceptance criteria to identify measurably expressed miRNA sequences, a repeated-measure ANOVA with pairwise comparisons between all passages and multiplicity adjustment was executed. A total of 12 miRNA sequences were found to be statistically significant (*p* < 0.05) between passage 3 and 7 where 8 of them were also statistically significant between passage 3 and passage 5, however, none were statistically significant between passage 5 and 7 (Table 1).

**Table 1:** Significant Differences in miRNA expression between Passages [P3, P5, P7]

#	miRNA Name	Sequence	P5/P3	P5/P3	P7/P3	P7/P3
			Adjusted	Fold	Adjusted	Fold
			*p*-value	Change	*p*-value	Change
1	hsa-miR-196b-5p	CCCAACAACAGGAAACTAC	0.0294	−1.05	0.0231	−1.05
2	hsa-miR-16-5p	CGCCAATATTTACGTGCTG	0.0464	−1.33	0.0334	−1.39
3	hsa-miR-1202	CTCCCCCACTGC	0.0294	1.39	0.0334	1.32
4	hsa-let-7 g-5p	AACTGTACAAACTACTACCT	0.0049	−1.19	0.0161	−1.15
5	hsa-miR-572	TGGGCCACCGCCG	0.0385	1.24	0.0335	1.26
6	hsa-miR-92a-3p	ACAGGCCGGGACAAGT	0.0334	−1.21	0.0385	−1.19
7	hsa-miR-638	AGGCCGCCACCCG	0.0464	1.28	0.0232	1.45
		AGGCCGCCACCCGC	0.0385	1.38	0.0310	1.49
8	hsa-miR-1915-3p	CCCGCCGCGTC	NS	NS	0.0385	1.32
9	hsa-miR-17-5p	CTACCTGCACTGTAAGC	NS	NS	0.0335	−1.22
10	hsa-miR-29b-1-5p	TCTAAACCACCATATGAAACCAG	NS	NS	0.0380	−1.13
11	hsa-miR-15b-5p	TGTAAACCATGATGTGCTG	NS	NS	0.0335	−1.42

*NS* Not Significant

A repeated measures ANOVA was performed on the 6 MSC donors that grew to passage 7. Positive fold change indicates upregulation in MSC expression at passage 7, while negative fold change indicates downregulation in MSC expression at passage 7

Eleven total miRNAs were found to be significantly different as two sequences differing by a single base pair, were identified as both miR-638. The miR-638 also exhibited the largest fold change between passages 3 and 7, 1.45 and 1.49, as well as fold changes between passages 3 and 5, 1.28 and 1.38. Using these robust methods, fold changes small as −1.05 could be resolved using the Agilent miRNA platform. A majority of the observed significant miRNAs had a mean magnitude of expression well above the negative control cutoff (Fig. 3e). Using unsupervised principal component analysis, which also included samples not used in the statistical analysis (127756 and PCBM1655), no distinct separation was observed between samples based on donor or passage (Fig. 3F). All samples regardless of donor and passage revealed one large cluster with a few samples trailing away from it. After applying supervised principal component analysis of the 12 significant miRNA sequences, the 61 samples display a parabolic shape in 3-dimensional space where passage 3 samples were observed on one side of the parabola with the passage 7 samples opposite it, and the passage 5 samples distributed through the vertex (Fig. 3g). One MSC line, PCBM1655, only grew to passage 3, but is seen distributed among passage 7 samples. Previous work of the MSC consortium has shown that this MSC line has a poorer capacity for proliferation and differentiation at passage 3, which is comparable to passage 7 samples of the other MSC lines. In comparing the lists of significantly different miRNAs between passages and the commonly expressed miRNAs in all 8 MSC donors, the 11 significant miRNAs were identified in both categories. Moreover, 60 of the commonly expressed miRNAs in all MSC donors did not exhibit any significant differences through cellular passaging.

### Confirmation by RT-qPCR

To further explore a MSC signature consisting of miRNAs, 19 miRNAs selected from Additional file 5: Table S3 were evaluated by RT-qPCR for detectable expression (C_q_ < 35). Based on the results of the microarray study, 16 miRNAs had detectable expression with 3 miRNAs being consensus miRNAs expressed in MSCs from the Clark et al., (let-7f, let-7i and miR-199a-5p) [49]. Another 3 miRNAs (miR-25-3p, miR-106b-5p, and miR-130b-3p) were not expressed in all MSC samples using the microarray platform and were evaluated by RT-qPCR to confirm our results. In our RT-qPCR studies, 2 of the known miRNAs, let-7f and let-7i, were observed to be expressed in all samples of both MSC sets, while miR-199a-5p was not (Additional file 8: Table S5). For the tested miRNAs not expressed from the microarray experiments, 2 miRNAs, miR-25-3p and miR-130b-3p, were observed to be expressed based on the RT-qPCR studies while 1 miRNA, miR-106b-5p, was not. Out of the 13 miRNAs being evaluated for new consensus miRNAs in MSCs, 8 miRNAs had detectable expression in both the microarray and RT-qPCR experiments (miR-22-3p, miR-27a-3p, miR-34a-5p, miR-193b-3p, miR-320b, miR-324-3p, miR-494, and miR-1260a). Five of the miRNAs (miR-320c, miR-320d, miR-365a-3p, miR-494, and miR-1305) did not have detectable expression when using the RT-qPCR platform.

**Fig. 4:**
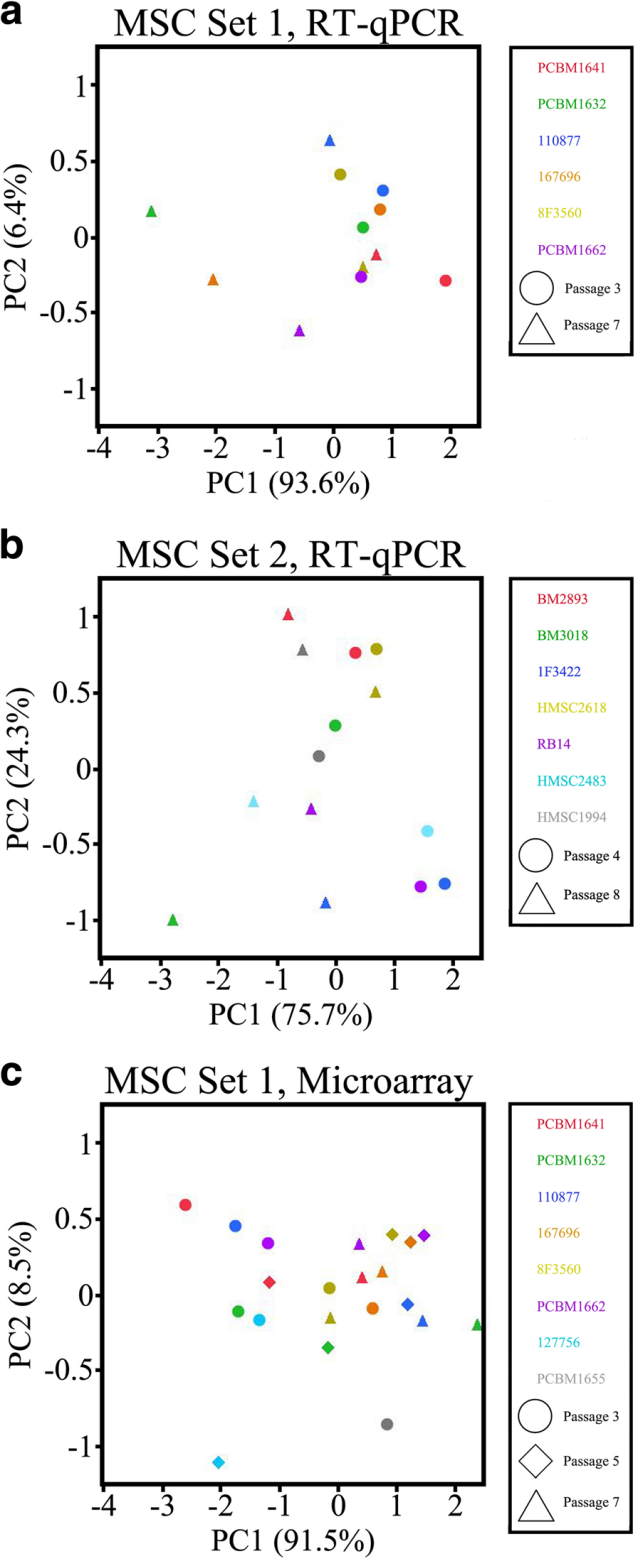
**a** Supervised principal component analysis of miR-572 and miR-638 for RT-qPCR data MSC set 1. **b** Supervised principal component analysis of miR-572 and miR-638 for RT-qPCR data MSC set 2. **c** Supervised principal component analysis of miR-572 and miR-638 for the microarray data (all donors and passages)

Based on the results of the miRNA microarray data, the 11 statistically significant miRNAs were evaluated by RT-qPCR. The expression of these miRNAs was evaluated in two sets of MSCs expanded from early to late passages (passage 3 vs 7 and passage 4 vs 8). In the first set of MSCs, only miR-572 and miR-638 were observed to be significantly (*p* < 0.05) upregulated 1.54 and 1.71 fold, respectively, at passage 7 compared to passage 3 (Additional file 8: Table S5). In contrast, for the second set of MSCs, 6 out of the 11 miRNAs (miR-196b-5p, miR-16-5p, miR-1202, miR-572, miR-638, and miR-15b-5p) evaluated for cellular aging were statistically significant (*p* < 0.05) between passage 4 and 8. miRNAs, miR-572 and miR-638 were significant in the second set of MSCs and upregulated at the later passage by 1.59 and 1.35, respectively. Supervised principal component analysis using these two miRNAs for both sets of MSC RT-qPCR data reveals some degree of separation between early and late passage MSCs (Figs. 4a–b). The results of the RT-qPCR experiments are comparable to the supervised principal component analysis of the miRNA microarray studies using the first set of MSCs (Fig. 4c). Passage 3 MSC samples are separated from passage 7 samples, while passage 5 samples are distributed amongst them. The two MSC samples, 127756 and PCBM1655, not used in the statistical analysis of the microarray data were evaluated in the principal component analysis. Sample 127756 at passage 3 clustered with passage 3 samples, while sample PCBM1655 at passage 3 (which did not expand beyond passage 3) was observed much further away from the passage 3 cluster.

The 19 miRNAs evaluated for detectable expression by RT-qPCR were also evaluated for statistical significance between early and late passages. For the first set of MSCs only 3 miRNAs (miR-324-3p, miR-494-3p, and miR-1260a) were observed to be statistically significant (*p* < 0.05) between passages 3 and 7. For the second set of MSCs, 7 miRNAs (let-7i, miR-25-3p, miR-106b-5p, miR-130b-3p, miR-199a-5p, miR-365a-5p, and miR-1260a) were statistically significant between passages 4 and 8. MiR-1260a was found to be significantly different between early and late passages for both MSC sets; however, it was upregulated at passage 7 for the first MSC set and downregulated at passage 8 for the second MSC set. A subsequent analysis was performed to evaluate whether the differences in miRNA expression between early and late passage was statistically different between the two sets of MSCs. Of the 30 miRNAs evaluated by RT-qPCR, 3 miRNAs, miR-320d, miR-365a-5p, and miR-1260a, were statistically significant (*p* < 0.05) between MSC set 1 and 2 (Additional file 8: Table S5). For all three miRNAs, the level of expression was greater at the earlier passage for MSC set 2, whereas the level of expression was greater at the later passage for MSC set 1. The observed differences in expression between the two MSC sets for the three miRNAs are indicative of the heterogenic miRNA expression that may have resulted from donor variability or expansion medium. Additionally, differences between miR-1260a between early and late passage were not observed for the microarray data analysis, therefore the expression of miR-1260a appears variable and potentially a false positive.

To compare the miRNA expression in MSCs with other cell types, four cell types of the mesoderm (2 dermal fibroblast lines, 1 osteoblast line, and 1 chondrocyte line) and four cancer cell types (embryonal carcinoma, adenocarcinoma, ovarian carcinoma, and pancreatic carcinoma) were evaluated for expression by the 30 aforementioned miRNAs (19 miRNAs to evaluate the magnitude of expression and 11 miRNAs to compare differences between early and late passage) using RT-qPCR. The goal of these experiments was to identify whether the expression of miRNAs expressed in MSCs were unique to them as compared to other cell types. In evaluating early passage MSCs (13 cell lines) against the 4 cell lines of the mesoderm lineage, 5 miRNAs (miR-15b-5p, miR-25-3p, miR-320d, miR-324-3p, and miR-494-3p) were observed to be significantly upregulated (*p* < 0.05) in the non-MSC lines. As previously mentioned, 45 miRNAs were observed to be significantly different in expression between cancer samples and early passage MSCs by microarray technology. Ten of the 45 miRNAs were evaluated by RT-qPCR experiments when comparing cancer cell lines to early passage MSCs. Similar to the microarray results, 5 of the 10 miRNAs (miR-196b-5p, let-7i-5p, miR-22-3p, miR-193b-3p, and miR-365a-5p) were significantly downregulated in the cancer cell lines compared to early passage MSCs. An additional 10 miRNAs were observed to be significantly different between the cancer cell lines and early passage MSCs for the RT-qPCR experiments, but not the microarray experiments.

## Discussion

MSCs are being studied for their potential function in regenerative medicine and immunosuppression applications. Their efficacy may be impacted by several factors including donor variation, tissue source, method of isolation and manufacturing process. The ability to accurately predict how a population of MSCs will behave remains a significant challenge to comprehending their potential safety and effectiveness. The International Society for Cellular Therapy (ISCT) characterized MSCs to have greater than 95% expression for CD73, CD90, and CD105, and less than 2% expression for CD14 or CD11b, CD34, CD45, CD79α or CD19 and HLA-DR [50]. Since the discovery of miRNAs, researchers have investigated miRNA expression in MSCs with the hope of establishing a consensus miRNA signature [49, 51–54]. Identifying markers such as miRNAs that can differentiate or serve as predictors of cellular passaging may be very helpful for further clinical applications. Using microarray technology and RT-qPCR, we identified miRNA markers that can distinguish MSCs from cancer cell lines. In addition, we identified 2 miRNAs (miR-572 and miR-638) that changed as a result of cellular passaging through in vitro expansion. The identification of two miRNAs as they change due to cellular expansion was based on: (1) two different platforms with uniquely different normalization techniques; (2) two different sets of MSCs expanded in different media; and (3) miRNA expression measured at different passages between the two sets of MSCs. Based on the statistical significance of miRNAs miR-572 and miR-638 across two different platforms and using two different sets of MSCs, these results are likely to be highly replicable. These two miRNAs exhibited upregulation at the later passage and may therefore be useful in determining when a population of MSCs begin to reach a higher passage number.

Our previous work explored the differences in gene expression as a result of cellular aging using the same set of MSCs [30]. We found 78 genes that were either significantly up or down regulated using gene expression microarray technology based on cellular passaging. Furthermore, through a number of different techniques, cellular proliferation was observed to significantly decrease at later passages for these MSCs, with similar results found by other investigators [30, 37, 55–57]. Previous research using the identical MSC lines has also shown differences in MSC differentiation by osteogenesis and adipogenesis, cell size, protein expression, immunosuppression, epigenetic profiling, and morphology [31–35, 38, 40]. These phenotypic changes may have corresponded with the gene expression differences observed between passages; therefore it is conceivable that some of these changes also correspond with the upregulation of miR-572 and miR-638 at later passages in MSCs.

miRNAs are intensely being evaluated as biomarkers for cancer in blood, urine and saliva. A number of studies have found miR-572 to be a potential marker when investigating tumor tissue compared to healthy tissue from various types of cancer such as colon cancer, ovarian cancer, basal cell carcinoma, nasopharyngeal carcinoma and renal cell carcinoma [58–63]. Zhang et al. observed that miR-572 was upregulated in ovarian cancer and likely corresponded with cancer progression and overall patient survival [63]. The study revealed that the expression of miR-572 inhibited the targets of suppressor of cytokine signaling 1 (*SOCS1*) and *p21* to promote cell proliferation and cell cycle progression of ovarian cancer cell lines, OVCAR3 and SKOV3. In another study by Wu et al., several additional ovarian cancer cells lines were evaluated where upregulation in the expression of miR-572 promoted ovarian cancer cell proliferation [62]. However in contrast, the gene, *PPP2R2C*, was identified as a target of miR-572 because its suppression by siRNA exhibited increased ovarian cell proliferation when miR-572 was also inhibited. In contrast, elevated miR-572 expression in MSCs at higher passages from our investigations corresponded with a lower capacity for proliferation.

MiR-638 which exhibited similar upregulation in expression at later passages as miR-572 in aging MSCs has been identified in a number of different carcinomas including colorectal, gastric, hepatocellular, nasopharyngeal, esophageal squamous cell, pancreatic adenocarcinoma, ovarian cancer and triple negative breast cancer [59, 64–73]. Many of these studies have evaluated the role and function of miR-638 in different cell types. In tumor derived cells and cell lines representative of colorectal carcinoma, leukemia, gastric carcinoma and triple negative breast cancer, the ectopic or overexpression of miR-638 inhibited cell proliferation and arrested the cell cycle in the G1 phase [69, 71, 72, 74, 75]. Similar findings were observed in non-cancerous cells such as human vascular smooth muscle cells where high expression of miR-638 inhibited proliferation and migration by targeting the NOR1/cyclin D pathway [76]. The general consensus that high expression of miR-638 inhibits cell proliferation is consistent with our findings where MSCs at higher passages exhibit higher expression of miR-638, but lower proliferation potential [30, 37].

The ability to confidently detect small differences in miRNA expression between groups lies in the reproducibility of the specific technology applied. Using similar methods as presented in López-Romero et al., we calculated the variability between identical sequences of the same samples for the microarray dataset. In López-Romero et al., two studies were used to demonstrate that low variability (standard deviation between replicates) was observed at low expression and as the magnitude of expression increased, the variability increased [42]. Our analysis exhibited the same phenomenon with both within chip and between chips variability; however, with slightly lower values at higher magnitudes of expression (Additional file 4: Figure S1). The within chip variability was lower than the between chip variability. The high reproducibility of the Agilent microarray platform is supported in a study by Mestdagh et al. where multiple miRNA expression platforms were evaluated for reproducibility, accuracy and specificity [77]. Based on the analysis in this article, it was reported that the Agilent microarray platforms had the highest reproducibility out of 12 platforms with the Qiagen miScript platform ranking ninth. For these reasons, the Agilent miRNA microarrays were well suited for detecting minute differences.

While the expression of miR-572 and miR-638 was consistently observed to be statistically upregulated at later passages, the number of other statistically significant miRNAs varied between the microarray and RT-qPCR platforms. Part of the discrepancy can be explained by the different normalizations methods where quantile normalization was used for the microarray platform and a group of reference markers were used for the Qiagen RT-qPCR platform. This point and the lower potential for reproducibility using the Qiagen RT-qPCR platform can explain part of the incongruity in results between both platforms. Additional knowledge regarding the level of miRNA expression in MSCs could be gathered using next generation sequencing methods, which could identify unknown miRNA sequences and quantify the total copy number.

Another point of interest is how the outcome of the RT-qPCR differed from each other and the microarray results. In our study, only 2 of the 11 miRNAs identified by microarray technology were found to be statistically significant for MSC set 1 by RT-qPCR, while 6 were found to be statistically significant for MSC set 2. In another study by Kundrotas et al. evaluating differences in miRNA expression due to cellular passaging, 33 miRNAs were observed to be statistically significant (*p* < 0.05) between early and late passage [78]. Of those 33 miRNAs identified in their study, only 3 miRNAs (miR-15b, miR-92a, and miR-29b-1-5p), were identical to the 11 miRNAs identified in our microarray study. In a different study by Kilpinen, researchers identified a total of 63 miRNAs using microarray technology that were significant between early and late passage bone marrow-derived MSCs isolated from both young (mean age 22.3) and old (mean age 76) donors [79]. Five miRNAs (miR-92a-3p, miR-1915-3p, miR-17-5p, miR-29b-1-5p & miR-15b-5p) from our list of 11 were identical to what this group observed for microarray. The group was only able to confirm the expression of 5 miRNAs by RT-qPCR technology when they excluded 2 donors from their dataset. Additionally, this research group also attempted to establish a MSC miRNA signature by identifying consistently expressed miRNAs where two on their list were miR-572 and miR-638. These results demonstrate that despite finding statistically significant results (*p* value less than a certain value), sometimes significance is specific to the set of MSCs under investigation unless the result can be observed across different MSC cell lines with similar experimental design. In our study, only miR-572 and miR-638 expression was observed to be significantly upregulated in two different sets of MSCs.

As indicated previously, identifying new miRNAs that are consistently expressed in MSCs derived from different donors may aid in establishing their identity. In a publication by Clark et al., 44 miRNAs are listed as consensus miRNAs expressed in MSCs, as determined by reviewing the scientific literature for miRNAs expressed in 2 or more microarray platforms or in one deep sequencing experiment [49]. Based on this list, we have identified 16 additional miRNAs that are commonly expressed by MSCs using 2 different platforms (microarray technology and RT-qPCR) and in 2 different sets of MSCs (Additional file 8: Table S5, “Yes” in column titled Median C_q_ < 35 in all MSC samples). Discrepancies were observed between platforms where some miRNAs were expressed by one platform, but not the other. When measuring the expression of miRNAs at the limit of detection, this inconsistency is likely to occur. One potential reason is the amount of the starting material used for the specific technology. In the microarray experiment, 100 ng was used per the manufacturer’s instructions, however, if a greater amount was used, there is a possibility for more miRNAs being expressed in all MSCs using the platform. Another potential source for causing an error is the amplification performed during the qPCR whereby a minimal amount of material is amplified until a signal is detected. Based on the robust experimental design and the methods, 16 miRNAs can be added to the Clark et al. list of miRNA commonly expressed for bone marrow derived MSCs.

The presented work has thoroughly shown that the expression of 2 miRNAs, miR-572 and miR-638, may be used to distinguish early and late passaged MSCs. Since cellular expansion is often essential for generating sufficient quantities for a cell-based therapy, these miRNA markers may be useful in establishing when an isolated population of MSCs is suitable for expansion. While these two miRNAs might be beneficial for evaluating the quality of MSCs during the manufacturing process, they are not the sole markers for evaluating differences between cellular passages as our previous work has explored using gene markers [30, 37]. Further work is needed to better characterize MSCs markers that can be linked to specific quality attributes of a therapeutic application.

## Conclusions

The current study identified 71 miRNAs commonly expressed out of 939 examined with microarray technology. Using RT-qPCR, 16 miRNAs were evaluated and observed to be consistently expressed in a second set of MSCs, which were not previously known. By microarray analysis, 11 miRNAs were also observed to be significantly different between early and late passages; however, only two miRNAs, miR-572 and miR-638, were observed to be upregulated at later passages. These results were based on using two different platforms to evaluate miRNA expression and two different sets of MSCs expanded in different media to different passages.

## Additional files

Additional file 1: Table S1. Cell Donor Characteristics. (DOC 53 kb)

Additional file 2: Table S2. Universal Human Reference RNA Cancer Cell Lines. (DOC 28 kb)

Additional file 3: Evaluation of Normalization Methods and Technical Variability Measurements. (DOC 28 kb)

Additional file 4: Figure S1. Boxplots representing the miRNA signal distribution of the A) raw data; B) AFE-TGS normalized data; and C) quantile normalized data. Kernel density plots for the distribution of the D) within chip technical variability; and E) between chip technical variability for both AFE-TGS and quantile normalized data. Mean expression per miRNA sequence for the quantile normalized data versus the F) within chip technical variability; and G) between chip technical variability. (JPEG 816 kb)

Additional file 5: Table S3. Common miRNA Sequences Expressed by 8 Different Donors at Three Passages. (XLS 38 kb)

Additional file 6: Figure S2. The mean absolute difference between cancer and MSC expression of samples versus the technical variability cutoffs of A) within chip; and B) between chips. C) Signal distribution of the negative controls used to determine the background cutoff. (JPEG 674 kb)

Additional file 7: Table S4. Significantly Different miRNA expression between Cancer [2 samples: Universal RNA & NT2] and MSCs [8 donors at passage 3]. Positive fold change indicates upregulation in cancer samples and negative fold change indicates down regulation in cancer samples. (XLS 33 kb)

Additional file 8: Table S5. Statistical comparisons of miRNAs between early and late passages for two MSC sets with RT-qPCR data. Statistical comparisons of miRNAs were also evaluated with Non-MSC (Mesoderm and Cancer) cell lines compared to MSCs at early passage. (DOC 71 kb)

Additional file 9: Table S6. Raw C_q_ values of the miRNA RT-qPCR. (XLSX 48 kb)

## Abbreviations

AFE-TGS: Agilent Feature Extraction Total Gene Signal
ANOVA: Analysis of Variance
EDTA: Ethylene diamine tetra acetic acid
MEM: Minimum Essential Medium
miRNA: MicroRNA
MSC: Multipotent Stromal Cell
RIN: RNA Integrity Value
RNA: Ribonucleic Acid
RT-qPCR: Reverse Transcription – Quantitative Polymerase Chain Reaction
