# Supplementary material for: MicroRNA expression in bone marrow-derived human multipotent Stromal cells
Source: BMC Genomics. 2017 Aug 11;18:605. doi: 10.1186/s12864-017-3997-7 (PMC5553681; doi:10.1186/s12864-017-3997-7)
Supplement: Supplementary file 1 — Cell Donor Characteristics. (DOC 53 kb) [file 12864_2017_3997_MOESM1_ESM.doc]

**Supplemental Table 1:** Cell Donor Characteristics

| **#** | **Donor** | **Cell Type** | **Sex** | **Age** | **Passage** | **Technology Used** | **Set** | **Company** |
| --- | --- | --- | --- | --- | --- | --- | --- | --- |
| 1 | PCBM1641 | MSC | Female | 23 | 3, 5, 7 | Microarray, RT-qPCR | Set 1 | AllCells |
| 2 | PCBM1632 | MSC | Male | 24 | 3, 5, 7 | Microarray, RT-qPCR | Set 1 | AllCells |
| 3 | 167696 | MSC | Female | 22 | 3, 5, 7 | Microarray, RT-qPCR | Set 1 | Lonza |
| 4 | 110877 | MSC | Male | 22 | 3, 5, 7 | Microarray, RT-qPCR | Set 1 | Lonza |
| 5 | 8F3560 | MSC | Female | 24 | 3, 5, 7 | Microarray, RT-qPCR | Set 1 | Lonza |
| 6 | PCBM1662 | MSC | Female | 31 | 3, 5, 7 | Microarray, RT-qPCR | Set 1 | AllCells |
| 7 | 127756 | MSC | Male | 43 | 3, 5 | Microarray |  | Lonza |
| 8 | PCBM1655 | MSC | Female | 47 | 3 | Microarray |  | AllCells |
| 9 | 1F3422 | MSC | Male | 39 | 4, 8 | RT-qPCR | Set 2 | AllCells |
| 10 | BM2893 | MSC | Male | 41 | 4, 8 | RT-qPCR | Set 2 | AllCells |
| 11 | BM3018 | MSC | Male | 39 | 4, 8 | RT-qPCR | Set 2 | AllCells |
| 12 | HMSC2483 | MSC | Female | Fetal | 4, 8 | RT-qPCR | Set 2 | Cell Applications |
| 13 | HMSC2618 | MSC | Female | Fetal | 4, 8 | RT-qPCR | Set 2 | Cell Applications |
| 14 | RB14 | MSC | Female | 20 | 4, 8 | RT-qPCR | Set 2 | Rooster Bio |
| 15 | HMSC1994* | MSC | Female | Fetal | 4, 8 | RT-qPCR | Set 2 | Cell Applications |
| 16 | HC1994* | Chondrocyte | Female | Fetal | 4 | RT-qPCR | Mesoderm | Cell Applications |
| 17 | HOb1994* | Osteoblast | Female | Fetal | 4 | RT-qPCR | Mesoderm | Cell Applications |
| 18 | NHDF271678 | Dermal Fibroblast | Female | 58 | 4 | RT-qPCR | Mesoderm | Cell Applications |
| 19 | NHDF293971 | Dermal Fibroblast | Male | 51 | 4 | RT-qPCR | Mesoderm | Cell Applications |
| 20 | NT2 | Embryonal carcinoma | Male | 22 |  | Microarray, RT-qPCR | Cancer | ATCC |
| 21 | OVCAR5 | Adenocarcinoma | Female | 60 |  | RT-qPCR | Cancer | ATCC |
| 22 | SKOV3 | Human ovarian carcinoma | Female | 64 |  | RT-qPCR | Cancer | ATCC |
| 23 | HS766T | Pancreatic carcinoma | Male | 46 |  | RT-qPCR | Cancer | ATCC |

* These cell types are from the same donor.
