# Supplementary material for: MicroRNA expression in bone marrow-derived human multipotent Stromal cells
Source: BMC Genomics. 2017 Aug 11;18:605. doi: 10.1186/s12864-017-3997-7 (PMC5553681; doi:10.1186/s12864-017-3997-7)
Supplement: Supplementary file 2 — Universal Human Reference RNA Cancer Cell Lines. (DOC 28 kb) [file 12864_2017_3997_MOESM2_ESM.doc]

**Supplemental Table 2**: Universal Human Reference RNA (from Agilent Technologies)

| Cell Line Derivations | |
| --- | --- |
| Adenocarcinoma, mammary gland | Melanoma |
| Hepatoblastoma, liver | Liposarcoma |
| Adenocarcinoma, cervix | Histiocytic lymphoma; macrophage; histocyte |
| Embryonal carcinoma, testis | Lymphoblastic leukemia, T lymphoblast |
| Glioblastoma, brain | Plasmacytoma; myeloma; B lymphocyte |
