# Supplementary material for: MicroRNA expression in bone marrow-derived human multipotent Stromal cells
Source: BMC Genomics. 2017 Aug 11;18:605. doi: 10.1186/s12864-017-3997-7 (PMC5553681; doi:10.1186/s12864-017-3997-7)
Supplement: Supplementary file 3 — Evaluation of Normalization Methods and Technical Variability Measurements. (DOC 28 kb) [file 12864_2017_3997_MOESM3_ESM.doc]

**Supplemental Methods**

Evaluation of Normalization Methods and Technical Variability Measurements

Two methods for normalized miRNA sequence signals by high-throughput analysis were compared. The two processed signals analyzed were the AFE-TGS and quantile methods. While the goal of the study was not to compare normalization techniques, it was imperative to determine an appropriate technique for processing the miRNA signal. The signal distribution of 10 representative microarrays (out of 61) yielded relatively uniform distributions for the AFE-TGS and quantile methods in comparison to the unprocessed data [**Supplemental Figures 1A – 1C**]. Both post-processing signals were evaluated for their ability to reduce variability between technique replicates. Within chip technical variability was calculated from the median standard deviation of replicate sequences of the 61 arrays. The between chip technical variability was calculated from the median standard deviation of replicate arrays of the 21 different MSC samples (passages 3, 5 and 7). These normalization methods produced normal distributions for both measures of technical variability, however, a larger distribution spread was observed for the AFE-TGS method [**Supplemental Figures 1D - 1E**]. Comparatively, the median within chip technical variability was 0.3896 and 0.0567; and the median between chip technical variability was 0.4745 and 0.0344 for AFE-TGS and quantile methods, respectively. Based on the literature regarding miRNA microarray normalization and the results presented, quantile normalization was used for further subsequent downstream analyses . An additional step was taken to evaluate the technical variability versus the mean miRNA sequence expression across all MSC samples [**Supplemental Figure 1F – 1G**]. These results indicate that technical variability increases with the magnitude of the signal and that the variability is greater between arrays of identical samples than within arrays of identical sequences.
