# Supplementary figures and images for: MicroRNA expression in bone marrow-derived human multipotent Stromal cells
Source: BMC Genomics. 2017 Aug 11;18:605. doi: 10.1186/s12864-017-3997-7 (PMC5553681; doi:10.1186/s12864-017-3997-7)

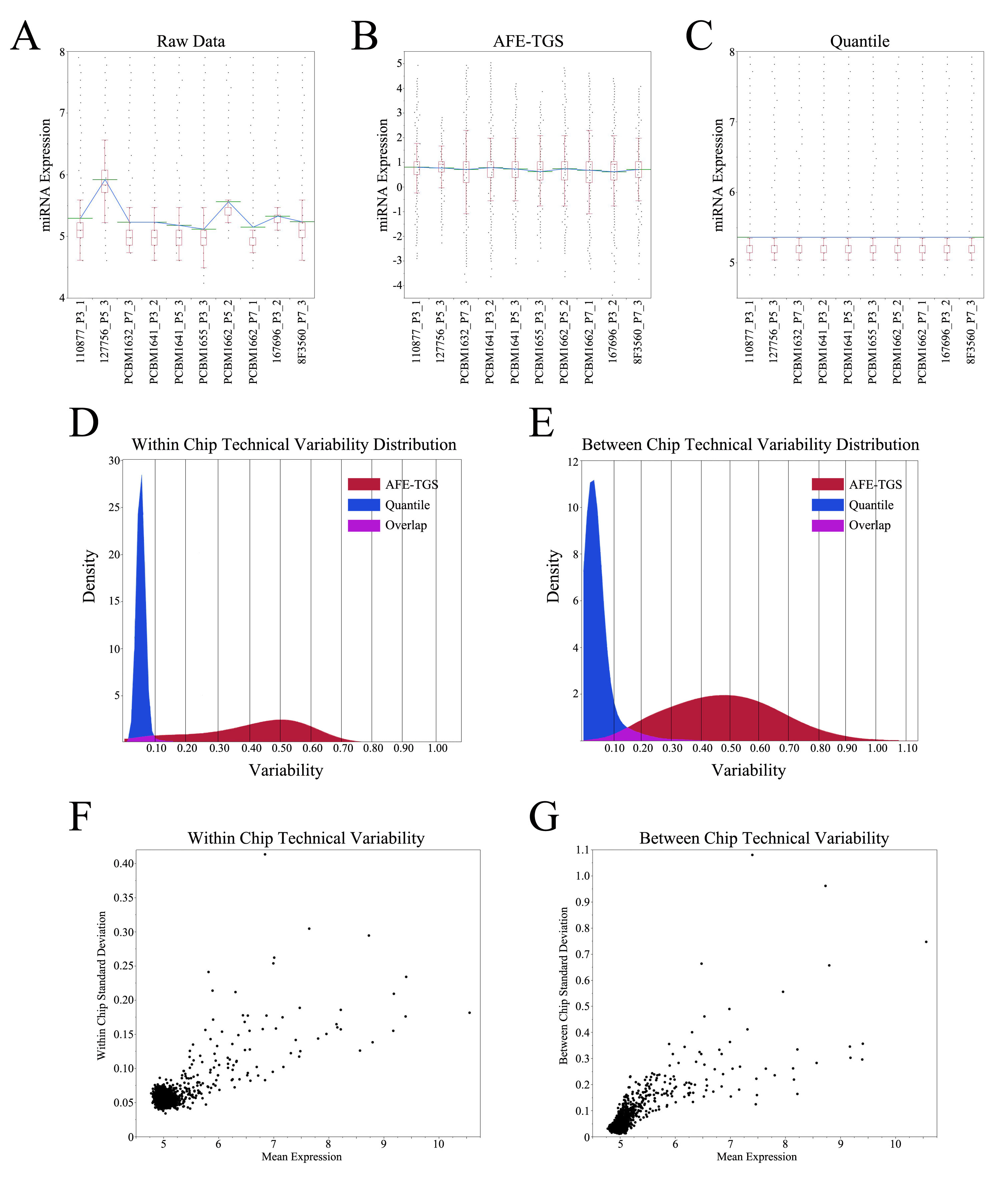

Supplement: Supplementary file 4 — Boxplots representing the miRNA signal distribution of the A) raw data; B) AFE-TGS normalized data; and C) quantile normalized data. Kernel density plots for the distribution of the D) within chip technical variability; and E) between chip technical variability for both AFE-TGS and quantile normalized data. Mean expression per miRNA sequence for the quantile normalized data versus the F) within chip technical variability; and G) between chip technical variability. (JPEG 816 kb) [file 12864_2017_3997_MOESM4_ESM.jpg]

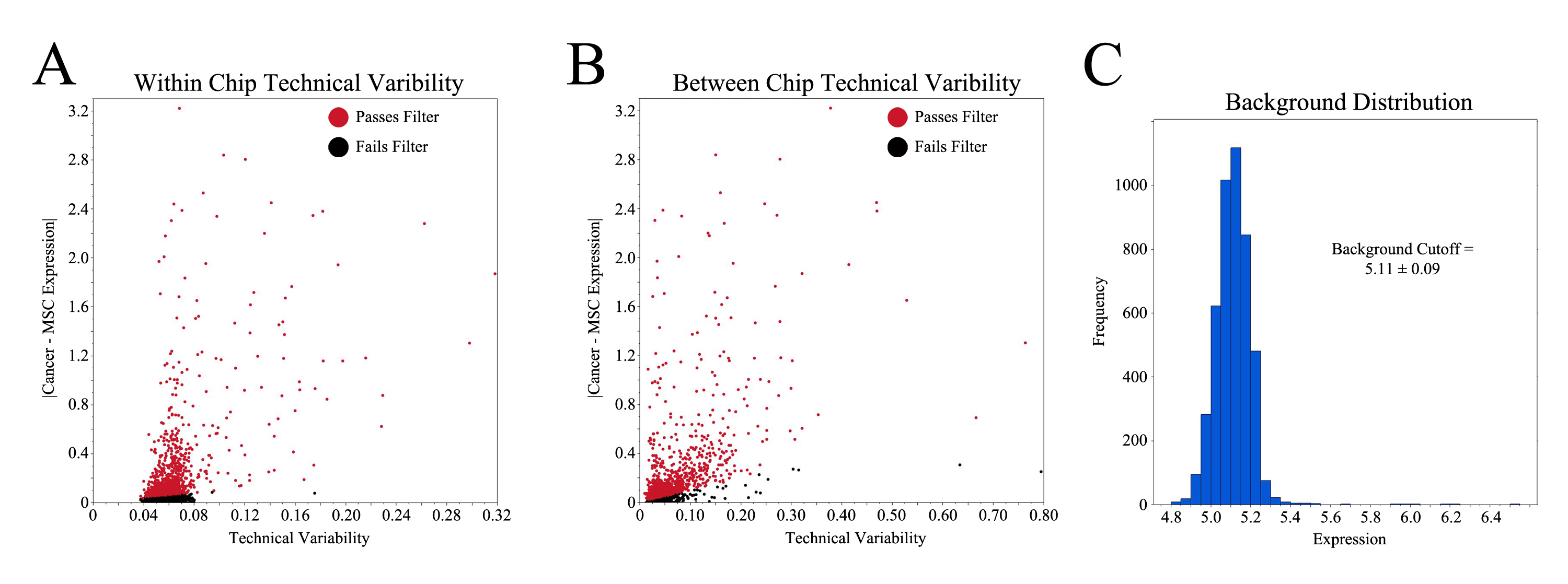

Supplement: Supplementary file 6 — The mean absolute difference between cancer and MSC expression of samples versus the technical variability cutoffs of A) within chip; and B) between chips. C) Signal distribution of the negative controls used to determine the background cutoff. (JPEG 674 kb) [file 12864_2017_3997_MOESM6_ESM.jpg]
