# Supplementary material for: MicroRNA expression in bone marrow-derived human multipotent Stromal cells
Source: BMC Genomics. 2017 Aug 11;18:605. doi: 10.1186/s12864-017-3997-7 (PMC5553681; doi:10.1186/s12864-017-3997-7)
Supplement: Supplementary file 8 — Statistical comparisons of miRNAs between early and late passages for two MSC sets with RT-qPCR data. Statistical comparisons of miRNAs were also evaluated with Non-MSC (Mesoderm and Cancer) cell lines compared to MSCs at early passage. (DOC 71 kb) [file 12864_2017_3997_MOESM8_ESM.doc]

**Supplemental TABLE 5**

| **MiRNA** | **MSC Set 1**  **P7 vs P3** | | **MSC Set 2**  **P8 vs P4** | | **Non-MSCs (Mesoderm)**  **vs MSCs** | | **Non-MSCs (Cancer)**  **vs MSCs** | | **Median C_q_ < 35, All Samples** | **Statistical Difference between MSC Sets 1 & 2** |
| --- | --- | --- | --- | --- | --- | --- | --- | --- | --- | --- |
|  | p-value | Fold Change | p-value | Fold Change | p-value | Fold Change | p-value | Fold Change |  |  |
| miR-196b-5p | NS | NS | 0.0248 | -2.19 | NS | NS | 1.6 x 10^-5^ | -11.47 | Yes | NS |
| miR-16-5p | NS | NS | 0.012 | -3.67 | NS | NS | NS | NS | Yes | NS |
| miR-1202 | NS | NS | 0.007 | -1.95 | NS | NS | NS | NS | Yes | NS |
| let-7g-5p | NS | NS | NS | NS | NS | NS | 0.0168 | -10.39 | Yes | NS |
| miR-572 | 0.042 | 1.54 | 0.004 | 1.59 | NS | NS | NS | NS | Yes | NS |
| miR-92a-3p | NS | NS | NS | NS | NS | NS | NS | NS | Yes | NS |
| miR-638 | 0.017 | 1.71 | 0.017 | 1.35 | NS | NS | 3.48 x 10^-6^ | -2.63 | Yes | NS |
| miR-1915-3p | NS | NS | NS | NS | NS | NS | 0.003 | -2.44 | Yes | NS |
| miR-17-5p | NS | NS | NS | NS | NS | NS | NS | NS | Yes | NS |
| miR-29b-1-5p | NS | NS | NS | NS | NS | NS | 0.0005 | -4.57 | Yes | NS |
| miR-15b-5p | NS | NS | 0.007 | -2.41 | 0.0002 | 2.77 | NS | NS | Yes | NS |
| let-7i-5p | NS | NS | 0.038 | -1.82 | NS | NS | 0.005 | -27.35 | Yes | NS |
| let-7f-5p | NS | NS | NS | NS | NS | NS | 0.0491 | -6.65 | Yes | NS |
| miR-22-3p | NS | NS | NS | NS | NS | NS | 3.61 x 10^-9^ | -9.80 | Yes | NS |
| miR-25-3p | NS | NS | 0.004 | -3.27 | 0.002 | 2.88 | NS | NS | Yes | NS |
| miR-27a-3p | NS | NS | NS | NS | NS | NS | 0.020 | -2.52 | Yes | NS |
| miR-34a-5p | NS | NS | NS | NS | NS | NS | 0.0033 | -4.99 | Yes | NS |
| miR-106b-5p | NS | NS | 0.0334 | -3.16 | NS | NS | NS | NS | No | NS |
| miR-130b-3p | NS | NS | 0.0123 | -2.65 | NS | NS | NS | NS | Yes | NS |
| miR-193b-3p | NS | NS | NS | NS | NS | NS | 0.0021 | -3.59 | Yes | NS |
| miR-199a-5p | NS | NS | 0.0239 | -8.75 | NS | NS | 0.0004 | -222.43 | No | NS |
| miR-320b | NS | NS | NS | NS | NS | NS | NS | NS | Yes | NS |
| miR-320c | NS | NS | NS | NS | NS | NS | NS | NS | No | NS |
| miR-320d | NS | NS | NS | NS | 0.002 | 2.66 | NS | NS | No | 0.030 |
| miR-324-3p | 0.0365 | 1.72 | NS | NS | 0.0028 | 1.86 | NS | NS | Yes | NS |
| miR-365a-5p | NS | NS | 0.003 | -2.13 | NS | NS | 1.64 x 10^-5^ | -4.78 | No | 0.007 |
| miR-494-3p | 0.032 | 1.31 | NS | NS | 0.032 | 1.58 | 1.13 x 10^-10^ | -32.07 | Yes | NS |
| mi-1207-5p | NS | NS | NS | NS | NS | NS | NS | NS | No | NS |
| miR-1260a | 0.022 | 1.40 | 0.028 | -1.82 | NS | NS | 0.007 | -2.45 | Yes | 0.009 |
| miR-1305 | NS | NS | NS | NS | NS | NS | NS | NS | No | NS |

Blue – MiRNAs selected for significant differences detected using microarray technology

Green – MiRNAs selected for measured expression in all donors/passage using microarray technology

NS – Not Significant
